# Nomenclatural novelties and notes in *Penstemon* (Plantaginaceae)

**DOI:** 10.3897/phytokeys.80.12962

**Published:** 2017-04-25

**Authors:** Craig C. Freeman

**Affiliations:** 1 R.L. McGregor Herbarium, Division of Botany, Biodiversity Research Institute, University of Kansas, Lawrence, Kansas 66047-3729, USA

**Keywords:** *Penstemon*, Plantaginaceae

## Abstract

Seven nomenclatural novelties in *Penstemon* (Plantaginaceae) are proposed for taxa that will be included in the forthcoming treatment of the genus in the Flora of North America North of Mexico series. Three additional novelties are made for Mexican taxa outside the flora area. *Penstemon xylus* A. Nelson is determined to be the correct name for the species heretofore called *P. tusharensis* N. Holmgren.

## Introduction

Field and herbarium studies of the genus *Penstemon* Schmidel (Plantaginaceae) for a treatment in the forthcoming Volume 17 of the Flora of North America North of Mexico (FNA) (Freeman in prep.) necessitate some nomenclatural changes for consistency in infraspecific rank, following FNA editorial guidelines. Three additional changes are made affecting two species that occur entirely in Mexico. Nomenclatural notes accompany some of the changes.

## Methods

Information about type specimens is based on protologues, major online nomenclatural indices (Tropicos - http://www.tropicos.org/; The International Plant Names Index - http://www.ipni.org/; JSTOR Global Plants – https://plants.jstor.org), herbarium specimens, and websites of individual herbaria (BR [http://www.botanicgarden.be/], CAS [http://www.calacademy.org/scientists/botany-collections], CM [http://www.carnegiemnh.org/botany/collection.html], F [https://www.fieldmuseum.org/node/5196], GH [http://huh.harvard.edu/pages/digital-resources], JE [http://herbarium.univie.ac.at/database/search.php], K [http://apps.kew.org/herbcat/navigator.do?_ga=1.106533571.1517483332.14897 82412], MICH [http://quod.lib.umich.edu/cgi/i/image/image- idx?xc=1;page=searchgroup;g=herb-ic], MIN [http://bellatlas.umn.edu/collections/index.php], MO [http://www.tropicos.org/], NY [http://sweetgum.nybg.org/science/vh/], RM [http://rmh.uwyo.edu/data/search.php], S [http://herbarium.nrm.se/search/specimens/], US [http://collections.nmnh.si.edu/search/botany/?ti=3]). Herbarium abbreviations follow *Index Herbariorum* (Thiers 2016, continuously updated, http://sweetgum.nybg.org/science/ih/). A digital image was examined for each specimen for which a barcode number is given in the type citation, followed by the source. Specimens examined for which no online digital images are available are not accompanied by barcode numbers.

## Nomenclatural novelties

### Penstemon cardinalis var. regalis

Taxon classificationPlantaeLamialesPlantaginaceae

(A.Nelson) C.C.Freeman stat. nov.

urn:lsid:ipni.org:names:77162263-1

#### Basionym.

*Penstemon regalis* A.Nelson, Amer. J. Bot. 21: 578–579. 1934; Penstemon cardinalis subsp. regalis (A.Nelson) G.T. Nisbet & R.C. Jacks.

#### Type.

USA. NEW MEXICO: near Carlsbad Cavern, Jun 1930, G. Convis 75 (holotype: RM, RM0004319 [JSTOR image]).

In the protologue, Nelson (1934) stated that the type was collected in May 1930. The date on the type specimen is June 1930.

### Penstemon crandallii var. procumbens

Taxon classificationPlantaeLamialesPlantaginaceae

(Greene) C.C.Freeman stat. nov.

urn:lsid:ipni.org:names:77162264-1

#### Basionym.

*Penstemon procumbens* Greene, Pl. Baker. 3: 23–24. 1901; Penstemon crandallii subsp. procumbens (Greene) D.D. Keck.

#### Type.

USA. COLORADO: Keblar Pass, in large mats on bare banks, 7 Aug 1901, C.F. Baker 733 (holotype: NDG, NDG45968 [JSTOR image]; isotypes: ARIZ, ARIZ-BOT-0005070 [JSTOR image], CAS, CAS0006571 [CAS website image], GH, GH00091383 [GH website image], JE, JE00002724 [JE website image], K, K000979692 [K website image], MO, MO-2217162 [MO website image], NY, NY00130478 [NY website image], RM, RM0004309 [JSTOR image], RM0004310 [JSTOR image], RSA, RSA0006207 [JSTOR image]), US, US00122356, [US website image], US011177691 [US website image]; probable isotypes: PH, PH00018550 [JSTOR image], RSA, RSA0006208 [JSTOR image]).

Label data on PH00018550 indicate the specimen was collected by Baker in 1900. Francis W. Pennell annotated the specimen as a probable isotype of Baker 733; David D. Keck annotated it as an isotype.

### Penstemon crandallii var. ramaleyi

Taxon classificationPlantaeLamialesPlantaginaceae

(A.Nelson) C.C.Freeman, comb. & stat. nov.

urn:lsid:ipni.org:names:77162265-1

#### Basionym:

*Penstemon ramaleyi* A. Nelson, Univ. Wyoming Publ. 3: 106. 1937.

#### Type.

USA. COLORADO. Saguache Co.: Villa Grove, 3 mi W, 9 Jul 1935; F. Ramaley & K.R. Johnson 15116 (holotype: RM, RM0004316 [JSTOR image]; isotypes: COLO, COLO00396432 [JSTOR image], K, K000979786 [K website image], MO).

### Penstemon eatonii var. exsertus

Taxon classificationPlantaeLamialesPlantaginaceae

(A.Nelson) C.C.Freeman stat. nov.

urn:lsid:ipni.org:names:77162266-1

#### Basionym.

*Penstemon exsertus* A. Nelson, Amer. J. Bot. 18: 438. 1931; Penstemon eatonii subsp. exsertus (A. Nelson) D.D. Keck.

#### Type.

USA. ARIZONA: In Salt River Canyon, on the Apache Trail, 4 May 1925, A. Nelson 10624 (holotype: RM, RM0004214 [JSTOR image]).

### Penstemon linarioides var. coloradoensis

Taxon classificationPlantaeLamialesPlantaginaceae

(A.Nelson) C.C.Freeman stat. nov.

urn:lsid:ipni.org:names:77162267-1

#### Basionym.

*Penstemon coloradoensis* A. Nelson, Bull. Torrey Bot. Club 26: 355. 1899; Penstemon linarioides subsp. coloradoensis (A. Nelson) D.D. Keck.

#### Type.

USA. COLORADO: Mancos, common on sage plains and foothills throughout the La Platas, 23 Jun 1898, C.F. Baker, F.S. Earle, and S.M. Tracy 70 (lectotype: RM, RM0004187 [JSTOR image]; isolectotypes: CAS, CAS0006620 [CAS website image], F, F0072589F [JSTOR image], GH, GH00091215 [GH website image], K, K000979702 [K website image], KSC , MICH, MICH1108038 [MICH website image], MO, MO-100230852 , NDG, NDG45904 [JSTOR image], NEB, NEB-v-0000619 [JSTOR image], NY, NY00091049 [NY website image], OS, OS0000264 [JSTOR image], RM, RM0004188 [JSTOR image], RSA, RSA0006119 [JSTOR image], RSA0006120 [JSTOR image], US, US00122246 [US website image]; probable isolectotype: NDG, NDG45695 [JSTOR image]).

In describing *Penstemon coloradoensis*, Nelson (1899) stated “*I have before me specimens from two collections made near Mancos, Colo., by Messrs. Baker, Earle, and Tracy, 1898 and distributed as P. caespitosus Nutt. Also from two collections by Professor Crandall, from Hotchkiss, Colo., 1892, and from Durango, 1898, both distributed as P. linarioides Sileri Gray.*” Nelson (1899) did not designate a holotype; the collections he cited are syntypes. Keck (1937) designated C.F. Baker, F.S. Earle, and S.M. Tracy 70 (RM) as the lectotype, of which there currently are two sheets, suggesting a second-step lectotypification might be necessary. Accession labels on the two sheets indicate they were received from the herbaria of F.S. Earle in 1899 (RM0004187; accession number 13703) and George E. Osterhout in 1938 (RM0004188; accession number 165282), subsequent to Keck’s lectotype designation. RM0004187 bears annotations by A. Nelson (*Penstemon coloradoensis* A. N. n. sp., without date), F.W. Pennell (*P. coloradoensis*, type, 1916), and D.D. Keck (P. linarioides A. Gray subsp. coloradoensis (A. Nelson) D.D. Keck, type, 1936). RM0004188, the later accession, was not annotated by Nelson, Pennell, or Keck. Whether Nelson had another sheet at RM that was lost, destroyed, or sent elsewhere, or had access to a sheet from some other herbarium, is not known. Of the two sheets currently at RM, only RM0004187 appears to have been there when Nelson described *P. coloradoensis* and later seen by Keck when he lectotypified the name. RM0004187 is taken as the lectotype, RM004188 is taken as an isolectotype, and no second-step lectotypification is needed. As an aside, the sheet at KSC is P. crandallii A. Nelson var. crandallii.

### Penstemon miniatus var. apateticus

Taxon classificationPlantaeLamialesPlantaginaceae

(Straw) C.C.Freeman stat. nov.

urn:lsid:ipni.org:names:77162268-1

#### Basionym.

*Penstemon apateticus* Straw, Bol. Soc. Bot. México 24: 42–43. 1959; Penstemon miniatus subsp. apateticus (Straw) Straw.

**Type**. MÉXICO. DISTRITO FEDERAL: Serrania de Ajusco, 10000 ft, Aug-Sep 1896, C.G. Pringle 6463 (holotype: CAS, CAS0003847 [JSTOR image]; isotypes: BR, BR0000008418234 [BR website image], CM, CM2018 [CM website image], F, F0072654F [F website image], GH, GH00091520 [GH website image], JE, JE00000356 [JE website image], MIN1001915 [MIN website image ; this specimen bears a second barcode, 189693, at the top of the sheet], MO, MO-155225 [MO website image], NDG, NDG45615 [JSTOR image], NY, NY00130560 [NY website image], PH, PH00020963 [JSTOR image], S, S10-20448 [S website image], US, US00122215 [US website image]).

### Penstemon miniatus var. townsendianus

Taxon classificationPlantaeLamialesPlantaginaceae

(Straw) C.C.Freeman, comb. & stat. nov.

urn:lsid:ipni.org:names:77162269-1

#### Basionym.

Penstemon apateticus subsp. townsendianus Straw, Bol. Soc. Bot. México 24: 44. 1959.

#### Type.

MÉXICO. CHIHUAHUA: collected in the Sierra Madres near Colonia Garcia, altitude 8000 ft, 1 Aug 1899, C.H.T. Townsend & C.M. Barber 211 (holotype: US, US00122217 [US website image]; isotypes: A, A00091591 [GH website image], F, F0072655F [F website image], GH, GH00091522 [GH website image], MO, MO-155224 [MO website image], NDG, NDG45616 [JSTOR image], NY, NY00130562 [NY website image], US, US01100658 [US website image]; probable isotype: NY, NY00130561 [NY website image]).

Straw (1959) designated US sheet number 568154 (=US00122217) as the holotype. He cited a second specimen at US as an isotype; that specimen is US sheet number 347089 (=US01100658). The label on NY00130561 does not bear the collector’s name or number; it was collected 5 mi SE of Colonia Garcia at an altitude of 7500 ft. Straw annotated the specimen as being Townsend and Barber 211.

### Penstemon pseudospectabilis var. connatifolius

Taxon classificationPlantaeLamialesPlantaginaceae

(A.Nelson) C.C.Freeman stat. nov.

urn:lsid:ipni.org:names:77162270-1

#### Basionym.

*Penstemon connatifolius* A. Nelson, Amer. J. Bot. 18: 437–438. 1931; Penstemon pseudospectabilis subsp. connatifolius (A. Nelson) D.D. Keck.

#### Type.

USA. ARIZONA: Steep banks, Apache Trail, 3 May 1925, A. Nelson 10314 (holotype: RM, RM0004190 [JSTOR image]; isotypes: NO, NO0109834 [JSTOR image], NY NY00091052 [NY website image], RSA, RSA0006126 [JSTOR image]).

### Penstemon spectabilis var. subinteger

Taxon classificationPlantaeLamialesPlantaginaceae

C.C.Freeman stat. nov.

urn:lsid:ipni.org:names:77162271-1

#### Basionym.

Penstemon spectabilis subsp. subinteger D.D. Keck, Amer. Midl. Naturalist 18: 817–818. 1937.

#### Type.

MÉXICO. BAJA CALIFORNIA: Santa Maria Plains, 15 mi S of Hamilton Ranch, plants 2-3 ft high, corolla bluish at lips, throat and tube with less blue than red, 8 Apr 1931, I.W. Wiggins 5207 (holotype: CAS, CAS003886 [CAS website image]; isotypes: CAS, CAS0003887 [CAS website image], F, F0072666F [F website image], GH, GH0091585 [GH website image], K, K000528837 [K website image], MICH, MICH1108051 [MICH website image], MO, MO-155229 [MO website image], NY, NY00130580 [NY website image], NY00130581 [NY website image], PH, PH00018576 [JSTOR image], RM, RM0004355 [RM website image], US, US00122381 [US website image]).

### Penstemon secundiflorus var. versicolor

Taxon classificationPlantaeLamialesPlantaginaceae

(Pennell) C.C.Freeman, comb. & stat. nov.

urn:lsid:ipni.org:names:77162272-1

#### Basionym.

*Penstemon versicolor* Pennell, Contr. U.S. Natl. Herb. 20: 358–359. 1920.

#### Type.

USA. Colorado. Pueblo Co.: clayey loam, mesa NE of Pueblo, ca 4700 ft, 5 Jun 1915, F. W. Pennell 5732 (holotype: NY, NY00029690 [NY website image]; isotypes: COLO, COLO00396481 [JSTOR image], US, US00122397 [US website image]).

## Nomenclatural note

### Penstemon xylus

Taxon classificationPlantaeLamialesPlantaginaceae

A. Nelson, Bot. Gaz. 31–32. 1902.

Penstemon caespitosus var. suffruticosus A. Gray in A. Gray et al., Syn. Fl. N. Amer. 2: 270. 1878 (non Penstemon suffruticosus Douglas ex Benth.); Penstemon tusharensis N.H. Holmgren, Brittonia 31: 106. 1979, nom. illeg.

#### Type.

USA. Utah. Beaver, 1877, Palmer s.n. (holotype: GH, GH00091198 [GH website image)]; probable isotype: ISC, ISC-v-0000867 [JSTOR image].

The correct name for this taxon at the rank of species has been the source of confusion. With the earlier name *Penstemon suffruticosus* Douglas ex Benth. blocking transfer of A. Gray’s varietal epithet *suffruticosus* at the rank of species, Rydberg (1901) published *P. suffrutescens* in an attempt to provide a substitute. However, in doing so, he flagged *P. suffrutescens* as a new species (sp. nov.). He also provided a brief diagnosis and cited a specimen (USA. Colorado. Ridgway, woods, altitude 7500 ft, 20 Jun 1895, F. Tweedy 170; holotype: NY, NY00130526 [NY website image]) different from that of Gray’s type for P. caespitosus var. suffruticosus. Rydberg’s name must be considered a validly published species name—not an avowed substitute for Gray’s name. As noted by Holmgren (1979), the specimen cited by Rydberg is referable to *P. crandallii*, where *P. suffrutescens* must be placed as a synonym.

Nelson (1902) published *Penstemon xylus* and clearly stated it was a new name (nom. nov.) for Gray’s P. caespitosus var. suffruticosus. Nelson cited a specimen (USA. Colorado. Sapinero, 1898, H.N. Wheeler 446 [COLO, COLO00396499 [JSTOR image], NY, NY00130556 [NY website image], RM, RM0004372 [JSTOR image]) that he believed was referable to A. Gray’s concept of P. caespitosus var. suffruticosus. However, it too is referable to *P. crandallii*. Holmgren (1979), believing that neither Rydberg’s nor Nelson’s name could be applied to Gray’s P. caespitosus var. suffruticosus because of the specimens cited by each author, published *P. tusharensis* as an avowed substitute. In spite of Nelson having cited a specimen that is not referable to P. caespitosus var. suffruticosus, *P. xylus* must stand as the avowed substitute for P. caespitosus var. suffruticosus at the rank of species, making *P. tusharensis* illegitimate and superfluous.

## Supplementary Material

XML Treatment for Penstemon cardinalis var. regalis

XML Treatment for Penstemon crandallii var. procumbens

XML Treatment for Penstemon crandallii var. ramaleyi

XML Treatment for Penstemon eatonii var. exsertus

XML Treatment for Penstemon linarioides var. coloradoensis

XML Treatment for Penstemon miniatus var. apateticus

XML Treatment for Penstemon miniatus var. townsendianus

XML Treatment for Penstemon pseudospectabilis var. connatifolius

XML Treatment for Penstemon spectabilis var. subinteger

XML Treatment for Penstemon secundiflorus var. versicolor

XML Treatment for Penstemon xylus
